# The Evolution of an Invasive Plant, *Sorghum halepense* L. (‘Johnsongrass’)

**DOI:** 10.3389/fgene.2020.00317

**Published:** 2020-05-14

**Authors:** Andrew H. Paterson, WenQian Kong, Robyn M. Johnston, Pheonah Nabukalu, Guohong Wu, William L. Poehlman, Valorie H. Goff, Krista Isaacs, Tae-Ho Lee, Hui Guo, Dong Zhang, Uzay U. Sezen, Megan Kennedy, Diane Bauer, Frank A. Feltus, Eva Weltzien, Henry Frederick Rattunde, Jacob N. Barney, Kerrie Barry, T. Stan Cox, Michael J. Scanlon

**Affiliations:** ^1^Plant Genome Mapping Laboratory, University of Georgia, Athens, GA, United States; ^2^School of Integrative Plant Science, Cornell University, Ithaca, NY, United States; ^3^The Land Institute, Salina, KS, United States; ^4^Department of Energy Joint Genome Institute, Walnut Creek, CA, United States; ^5^Department of Genetics & Biochemistry, Clemson University, Clemson, SC, United States; ^6^International Crops Research Institute for the Semi-Arid Tropics, Bamako, Mali; ^7^Genomics Division, National Institute of Agricultural Sciences, Jeonju, South Korea; ^8^College of Agricultural and Life Science, University of Wisconsin-Madison, Madison, WI, United States; ^9^School of Plant and Environmental Sciences, Virginia Tech, Blacksburg, VA, United States

**Keywords:** invasion biology, polyploidy, evolutionary novelty, weed, crop, rhizome, perennial

## Abstract

From noble beginnings as a prospective forage, polyploid *Sorghum halepense* (‘Johnsongrass’) is both an invasive species and one of the world’s worst agricultural weeds. Formed by *S. bicolor* x *S. propinquum* hybridization, we show *S. halepense* to have *S. bicolor*-enriched allele composition and striking mutations in 5,957 genes that differentiate it from representatives of its progenitor species and an outgroup. The spread of *S. halepense* may have been facilitated by introgression from closely-related cultivated sorghum near genetic loci affecting rhizome development, seed size, and levels of lutein, a photochemical protectant and abscisic acid precursor. Rhizomes, subterranean stems that store carbohydrates and spawn clonal propagules, have growth correlated with reproductive rather than other vegetative tissues, and increase survival of both temperate cold seasons and tropical dry seasons. Rhizomes of *S. halepense* are more extensive than those of its rhizomatous progenitor *S. propinquum*, with gene expression including many alleles from its non-rhizomatous *S. bicolor* progenitor. The first surviving polyploid in its lineage in ∼96 million years, its post-Columbian spread across six continents carried rich genetic diversity that in the United States has facilitated transition from agricultural to non-agricultural niches. Projected to spread another 200–600 km northward in the coming century, despite its drawbacks *S. halepense* may offer novel alleles and traits of value to improvement of sorghum.

## Introduction

Cytological, morphological (Celarier, 1958; Doggett, 1976), and molecular data (Paterson et al., 1995) suggest that tetraploid *Sorghum halepense* (2n = 40) arose as a naturally occurring hybrid between *S. bicolor* (2n = 20), an annual, polytypic African species which includes cultivated sorghum; and *S. propinquum* (2n = 20), a perennial southeast Asian native of moist habitats. While a firm estimate of its antiquity is lacking, *S. propinquum* is thought to have shared ancestry with *S. bicolor* ∼1–2 million years ago (Feltus et al., 2004), roughly circumscribing the maximum age of *S. halepense*. Occasionally used as forage and even food (seed/flour), *S. halepense* has spread in post-Columbian times from its hypothesized west Asian center of origin across much of Asia, Africa, Europe, North and South America, and Australia. Its establishment in the U.S. is probably typical of its spread to other continents, being introduced intentionally as a prospective forage and unintentionally as a contaminant of seedlots (McWhorter, 1971). However, while sorghum largely remained confined to cultivation, *S. halepense* readily naturalized and has spread across much of North America, both to agricultural and non-agricultural habitats (Sezen et al., 2016) – suggesting capabilities for adaptation well beyond those of sorghum.

Its common name thought to be a misnomer [the eponymous Col. Johnson may have obtained propagules from his wife’s family, who accidentally introduced it to South Carolina shortly after the Revolutionary War (Tellman, 1996)], ‘Johnsongrass’ has the rare distinction of being both a noxious weed in 20 U.S. states and an invasive species in 16 (Quinn et al., 2013). With at least 24 herbicide-resistant biotypes now known (Heap, 2012), Johnsongrass appears likely to become even more problematic in the future. For example, a glyphosate resistant biotype discovered in Argentina in 2002 covered 10,000 ha by 2009 (Binimelis et al., 2009). Its ability to cross with sorghum despite a ploidy barrier (reviewed in Warwick and Black, 1983; Tang and Liang, 1988) makes Johnsongrass a paradigm for the dangers of crop ‘gene escape,’ and restricts deployment of many transgenes that could reduce the cost and increase the stability of sorghum production.

Here, we integrate several diverse data types to elucidate the evolution of *S. halepense*, its invasiveness as exemplified by rapid spread across the United States in post-Columbian times, and the roles of polyploidy and interspecific hybridity in distinctive features of its growth and development. As the first surviving polyploid in its lineage in ∼96 million years (Paterson et al., 2009; Wang et al., 2015), *S. halepense* may also open new doors to sorghum improvement, with synergy between gene duplication and interspecific hybridity nurturing the evolution of genes with new or modified functions (Ohno, 1970).

## Materials and Methods

### Genome Size Determination

*Sorghum halepense* genome size is an average for five accessions based on flow cytometry performed on a fee for service basis under the supervision of K. Arumuganathan, Benaroya Research Institute, using published methods (Arumuganathan and Earle, 1991).

### Resequencing

*Sorghum halepense*, *S. propinquum*, *S. timorense* and representatives of each of the wild *S. bicolor* races (*S. bicolor* ssp. drummondii, SRP116974; *S. bicolor* ssp. verticilliflorum race aethiopicum, SRP116975: *S. bicolor* ssp. verticilliflorum race arundinaceum, SRP116973; *S. bicolor* ssp. verticilliflorum race verticilliflorum, SRP116978: *S. bicolor* ssp. verticilliflorum race virgatum SRP116940) were sequenced using standard methods implemented at the US Department of Energy Joint Genome Institute, as part of a larger project including 27 genomes and 39 transcriptomes in total. From each accession, 76-bp paired-end reads were aligned to the *Sorghum bicolor* reference genome (v1.4) using BWA version 0.5.9 (Li and Durbin, 2009). Multiple-sample SNP calling was performed using the mpileup program in the samtools package and bcftools (Li et al., 2009). Reads with mapping quality score > = 25 and base quality > = 20 are used for SNP calling. Raw SNPs are further filtered according to read depth distribution to avoid paralog contamination and low coverage regions. Each accession’s genotype is calculated using maximum likelihood estimation using reads with coverage between 4 and 30X. The genotype with the largest likelihood is assigned to each individual. SNPs with allele frequency > = 0.01 are used for downstream analysis.

As tandem genes are often recently derived and share high sequence similarity, they can complicate short read alignment and introduce ‘false SNPs’ from paralogs. To address this, the coverage of genomic reads (not including transcriptome data) was examined for every tandem gene in the sorghum genome. The average coverage of the whole genome across the 27 genomes studied is about 553X. There were 31 tandem genes with more than twice the genome coverage (1100X), of which 7 have coverage more than 2500X (ranging up to 7500X). A total of 14 of the 31 high coverage tandem genes have SNPs called, and were removed from further analysis.

### SNP Inference

To identify *S. halepense* SNPs, reads from *S. halepense* were aligned to the reference *S. bicolor* genome by BWA and SNPs determined with nucleotide groups for each reference *S. bicolor* genomic position by an in-house script. False positive *S. halepense* SNPs for each position of the reference *S. bicolor* genome were inferred and removed, based on three criteria: (i) if the top two nucleotide groups are the same as reference *S. bicolor* and *S. propinquum*, respectively, there are no false positive SNPs; (ii) if read depth of an SNP is 1 (noting the average 14X coverage of the *S. halepense* genome), a false positive was inferred; (iii) if *p*-value calculated by the Fisher exact test for the actual and theoretical read depths (bicolor:propinquum is 1:1), is less than 0.1, a false positive was inferred. The full SNP table with the reference *S. bicolor*, *S. propinquum*, and *S. halepense* SNPs as well as wild *S. bicolor* and *S. timorense* SNPs determined with total RNA and genomic DNA, respectively, against the reference *S. bicolor* genome, is provided (Supplementary Table 1). Classifications of duplicated genes into paralogs versus homologs followed the *S. bicolor* reference genome (Paterson et al., 2009).

### Gene Functional Enrichment Analysis

Arabidopsis GO-slim gene annotation was used for function enrichment analysis. GO-slim terms are assigned to sorghum genes based on sequence similarity inferred from best blastp hit. Binomial distribution based on the proportion of a GO-slim term among all annotated genes in the sorghum genome is used as the null distribution. Test significance threshold is defined as *p* < 0.05, unless specified otherwise.

### Functional Impact of SNPs

A customized script is used to map SNPs to the *Sorghum bicolor* gene model version 1.4. Striking SNPs are identified as those mapped to coding regions, splicing sites, stop codons and transcription initiation sites. The functional impact of non-synonymous SNP is assessed based on the evolutionary conservation profile of amino acids. Orthologous groups of protein sequences from 30 plant species are constructed using OrthoMCL. Protein sequences from each orthologous group are aligned using Clustalw2 (Larkin et al., 2007). Non-synonymous SNPs are mapped to the alignment of the corresponding orthologous group and a ‘functional impact score’ is calculated with a modified entropy function (Reva et al., 2011):

$$\begin{array}{c} S_{i}\left(\alpha \to \beta \right)=\{-\ln\frac{n_{i}\,\left(\beta \right)+1}{n_{i}\,\left(\alpha \right)}P_{c}\;ifn_{i}\left(\beta \right)+1<n_{i}\left(\alpha \right)\\ -\ln\frac{n_{i}\,\left(\beta \right)+1}{n_{i}\,\left(\alpha \right)}\,\left(1-P_{c}\right)\;i\,f\,n_{i}\,\left(\beta \right)+1>n_{i}\,\left(\alpha \right) \end{array}$$

where, α, β are 20 amino acid residues and gaps, *n_*i*_(*α) is the number of occurrences of residue α in an alignment column *i*. *n_*i*_(*β) is the number of occurrences of an alternative residue β in the column *i*. *Pc* is the probability of occurrence of the most common residue in the alignment column *i*. Si is the function index score, a measure of functional impact of a mutation on protein function. The significance threshold of *S*_*i*_ is determined at the FDR = 0.01.

### Survival of Cold or Dry Seasons

Survival of cold (temperate) or dry (Mali) seasons was based on single plants (*SbxSh* F2), or at least some survival within progeny rows of about 5 (*SbxSp* RILs) or 10 plants (*SbxSh* BC1F2; F3). Methods for determining rhizome numbers and distances from the originating crown are as cited (Kong et al., 2015). Flowering time was based on the average number of days from planting to flowering of either single plants (*SbxSh* F2) or the first five plants in a plot, and vegetative biomass was determined at the end of the growing season (after frost) by harvesting all tissue >2 cm above the ground for entire plots, separating inflorescences from vegetative tissues, drying to stable mass, and determining dry tissue mass. Heritabilities were calculated from F_2_–F_3_ regression (*S. bicolor* x *S. halepense* F_2_), or variance component analysis [*S. bicolor* x *S. halepense* BC_1_F_2_; *S. bicolor* x *S. propinquum* RILs (Kong et al., 2015)].

A logistic regression was performed using dry-season survival by each genotype as the response variable (0 or 1) and the distance between rhizome derived shoots and the crown based data from Athens, GA in 2013 (‘Dist’), as the explanatory variable. The model is:

l⁢n⁢(p/(1-p))=-2.1358+0.2339×D⁢i⁢s⁢t,

where p is the probability of survival. With 1 cm increments in Dist, the probability of survival increased by 3%.

### Laser Microdissection RNA-seq (LM RNA-seq)

**FIGURE 1:**
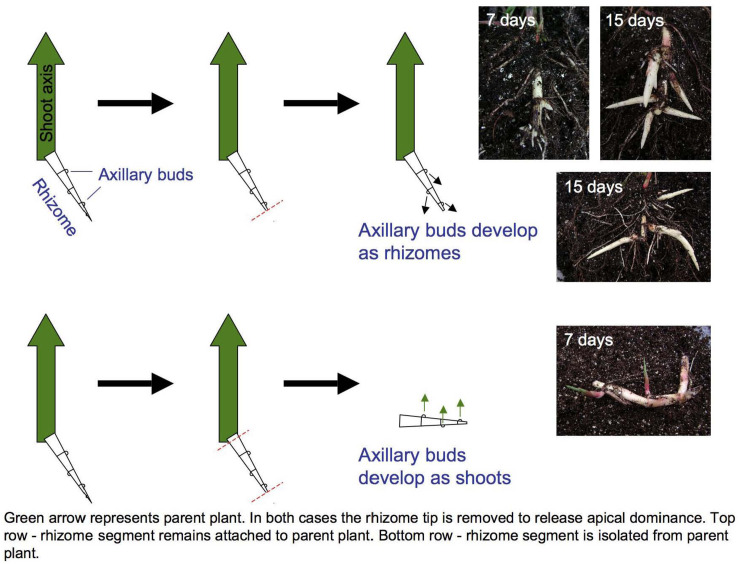
Excising primary rhizome tips induces *Sorghum halepense* axillary bud growth. Buds on rhizomes attached to the parent shoot (large green arrows) develop as secondary rhizomes (above), whereas buds on excised rhizomes develop as leafy shoots (below). Mechanical excision ensured the identity and equivalent developmental staging of shoot apices selected for transcriptional profiling.

LM RNA-seq was used to compare transcript accumulation in the shoot apices of buds induced to develop as either secondary rhizomes or leafy shoots (Figure 1). The meristem plus two youngest leaf primordia were microdissected from transverse sections. Two replicates of each meristem type were collected, with 5 meristems per replicate. LM, RNA extraction and amplification, cDNA library preparation and Illumina sequencing were performed as described (Takacs et al., 2012). LM RNA-seq reads are archived under NCBI BioProject ID PRJNA356741.

### Specificity of Gene Expression

*Sorghum halepense* RNAseq FASTQ files were preprocessed with (Bolger et al., 2014) (0.22) and assembled into a transcriptome reference assembly using Trinity (Haas et al., 2013) (r06-08-2012; –kmer_method jellyfish). Transcript mapping to the reference sorghum genome, and differential gene expression was performed with TopHat (Trapnell et al., 2009) (v2.0.3), Bowtie2 (Langmead and Salzberg, 2012) (2.0.0.6), Samtools (Li et al., 2009) (0.1.18.0), and Cufflinks (Trapnell et al., 2012) (2.0.0). From the FPKM values in Supplementary Table 4, three gene lists were created: (1) Significant differentially expressed genes between shoot and rhizome; (2) Genes ON in rhizome and OFF in Shoot; (3) Genes ON in shoot and OFF in rhizome. The rank order of differentially expressed genes was based on the cuffdiff test statistic (Trapnell et al., 2010), which was very closely correlated with the fold change in gene expression (Supplementary Table 5). To annotate these lists, the most recent *S. bicolor* reference genome annotation (v3.1) was downloaded from Phytozome v11.0 and annotation labels for GO, KEGG, and PFAM and were assigned to the *S. halepense* transcripts via homolog mapping with BLASTN. Genes were categorized as ON if there was any expression detected (FPKM > 0), and OFF if the FPKM value was zero. Term enrichment was performed using the David (da Huang et al., 2007) method re-implemented in a Perl script where the gene background was limited to a non-redundant list of *S. bicolor* transcripts that mapped to the Trinity transcript IDs from Supplementary Table 4.

### Correspondence of Sorghum QTLs to Introgression Hotspots

Non-random correspondence of sorghum QTLs from a published database (Zhang et al., 2013) with seven chromosomal ‘hotspots’ for introgression of sorghum alleles in five geographically diverse US *S. halepense* populations (Morrell et al., 2005) was determined using the hypergeometric probability distribution function, as described (Feltus et al., 2006).

## Results

### Mosaic Genome of *S. halepense*, With *S. bicolor* Enriched Allele Composition

While its 2.73 ± 0.08 pg/2C genome size closely approximates the sum of those of its progenitors, *S. halepense* has *S. bicolor* enriched allele composition (Table 1). To investigate its allele composition, we resequenced tetraploid *S. halepense* accession Gypsum 9 (SRX142088) to a depth of 9.7 Gb, ∼14X coverage of the *S. bicolor* reference genome and conferring ∼95% confidence of detecting *S. halepense* alleles present in as little as one copy. Assuming that tetraploid *S. halepense* has twice the 41,800,275 bp coding DNA sequence (CDS) length of the *S. bicolor* reference genome (Paterson et al., 2009) (Table 1 and Supplementary Table 1), 99.4% of *S. halepense* CDS nucleotides match those of representatives of ‘eusorghum (Kellogg, 2013; Hawkins et al., 2015)’ progenitor species *S. bicolor* (Paterson et al., 2009) and *S. propinquum* (SRX030701-03), and an outgroup *Sorghum* [*Sarga* (Hawkins et al., 2015)] *timorense* (SRX124552). Among the remaining 500,303 polymorphic nucleotide positions (Table 1, patterns 1–15), 10.9% match the *S. bicolor* reference but differ from *S. propinquum* (patterns 2, 3, 8, 9), and 6.6% match *S. propinquum* but not *S. bicolor* (patterns 6, 7, 12, 14). The *S. bicolor* and *S. propinquum* alleles were frequently interleaved along *S. halepense* chromosomes, indicating extensive homogenization (Kong, 2017). This is consistent with largely normal pairing and recombination between *S. bicolor* and *S. propinquum* diploids that is well-known from genetic studies (Chittenden et al., 1994; Paterson et al., 1995; Kong et al., 2015), and with segregation patterns in two interspecific (*S. bicolor* x *S. halepense*) BC_1_F_1_ populations that suggest a mixture of disomic and polysomic inheritance (Kong, 2017). While our analysis includes an outgroup and compares taxa separated by a minimum of 1–2 million years, some differences among these taxa presumably reflect within-species divergence.

**TABLE 1:** Coding DNA sequence polymorphism patterns among *Sorghum halepense*, its progenitors *S. propinquum* and wild *S. bicolor*, an elite domesticated *S. bicolor*, and the outgroup *S. timorense* (x indicates sequence divergence, o indicates correspondence).

Pattern	Number	Percentage (excluding pattern 16)	*S. timorense*	*S. propinquum*	*S. bicolor* (wild forms)	*S. bicolor* (reference genome)	Hypothesized interpretation
1	335775	67.1	X	X	X	X	*Novel SH alleles*
2	1259	0.3	X	X	X	O	*Possible introgression from cultivated S. bicolor*
3	1897	0.4	O	X	X	O	*Same as #2*
4	29096	5.8	O	X	X	X	*Alleles reflecting para-eusorghum divergence*
5	1250	0.2	X	O	X	O	*No clear inference*
6	23776	4.8	X	O	X	X	*SP-specific alleles*
7	7160	1.4	O	O	X	X	*Same as #6*
8	37532	7.5	O	X	O	O	*Wild SB-specific alleles*
9	13691	2.7	X	X	O	O	*Same as #8*
10	1652	0.3	O	X	O	X	*Wild SB-specific alleles changed by domestication*
11	5527	1.1	X	X	O	X	*Same as #10*
12	1221	0.2	X	O	O	X	*Ancestral eusorghum alleles changed by domestication*
13	34862	7.0	X	O	O	O	*Ancestral eusorghum alleles*
14	675	0.1	O	O	O	X	*Ancestral para-eusorghum alleles changed by domestication*
15	4930	1.0	O	O	X	O	*No clear inference*
16*	83100247		O	O	O	O	*Conserved ancestral para-eusorghum alleles*

	*Calculation of the number of pattern 16						

	41800275		(a) Total CDS length of *S. bicolor* (improved forms)				
	83600550		(b) Inferred total CDS length of *S. halepense* (2X CDS length of *S. bicolor*)				
	500303		(c) Number of alleles which were not strictly conserved (sum of patterns 1 to 15)				
	83100247		(d) b-c = number of pattern 16				

### *S. halepense* Is Richly Polymorphic

Despite a presumed genetic bottleneck during polyploid formation, *S. halepense* is richly polymorphic. A survey of 182 genetically-mapped restriction fragment length polymorphism (RFLP) loci found 18 *S. halepense* or *‘Sorghum x almum*’ (*S. bicolor* x *S. halepense* hybrid) genotypes to average 6.13 alleles per locus, versus 3.39 for a worldwide sample of 55 landrace and wild sorghum accessions and 1.9 for 16 F1 hybrid sorghums from eight commercial breeding programs (Morrell et al., 2005).

While some apparently novel alleles in the draft genome (Table 1) may reflect intraspecific polymorphism, a remarkable 67.1% of CDS polymorphisms differentiate *S. halepense* from representatives of both putative progenitor species and the outgroup *S. timorense* (Table 1, pattern 1). The functional impact of these non-synonymous single-nucleotide polymorphisms (SNPs) was assessed by comparison to an evolutionary conservation profile of amino acids from orthologous genes in a panel of diverse plant species, calculating a ‘functional impact score’ using a modified entropy function (Reva et al., 2011) – 8738 SNPs with high inferred functional impact score’ (*S*_*i*__;_ see section “Materials and Methods”) suggest important consequences for protein function in 5957 *S. halepense* genes (Supplementary Table 2). SNPs causing premature protein translation termination (5981 in 4459 genes) are most abundant, followed by loss of stop codons (2521 in 2016 genes) and loss of translation initiation site (236 in 227 genes). These functionally important mutations are significantly enriched in plasma membrane genes with kinase activity, suggesting changes in environmental sensing and associated intracellular processes such as cell differentiation and metabolism (Supplementary Table 3).

### Rhizomes Are Important to Survival of Both Cold Seasons and Dry Seasons

Rhizomes, subterranean stems that can comprise 70% of its dry weight (Oyer et al., 1959), are a key link between morphology and ecology of *S. halepense*. Rhizome growth of polyploid *S. halepense* transgresses that of its rhizomatous diploid progenitor, *S. propinquum*. We conducted a field trial in Bogart, GA (33.9° N) during 2012-3 of widely spaced (1 m between plants and rows) tetraploid F2 progeny from a cross between *S. bicolor* BTx623 and *S. halepense* Gypsum 9E (*SbxSh*); side by side with plots of 161 diploid recombinant inbred lines from a cross between BTx623 and *S. propinquum* (*SbxSp*; 5 plants per line, spaced 0.3 m between plants and 1 m between rows and plots) (Kong et al., 2015). *SbxSh* progeny had a higher frequency of rhizome-derived shoots emerging from the soil (37.6%), larger average number of rhizomes producing above-ground shoots (0.77), and greater distance of rhizome-derived shoots from the crown (11.97 cm) than *SbxSp* (30%, 0.32, 7.5). Rhizome number showed heritabilities of 0.077 (F_3_–F_2_ regression) and 0.34 (variance component analysis of BC_1_F_2_ families) in *SbxSh* and 0.44 in *SbxSp* (by variance component analysis).

Rhizomatousness is closely related to the ability of *S. halepense* to overwinter in the temperate United States. In the Bogart, GA field trial, 139 (58.9% of) *SbxSh* progeny showed regrowth after overwintering, while there was no survival of *SbxSp* in 2012-3 or in two additional years. Moreover, in *SbxSh* BC_1_F_1_-derived BC_1_F_2_ families (*n* = 246) grown in 3 m plots with two replications following conventional sorghum recommendations, those with rhizomes had significantly higher frequencies of survival than those lacking rhizomes (Table 2). The advantage of rhizomes was observed both in harsh winters (2013-14, with five periods below 20 F, reaching a low of 5.8 F^1^) and mild winters (2014-15, with only two periods below 20 F, reaching a low of 10.2 F) in Bogart GA. Survival in Salina, KS among replica plots of the same BC_1_F_2_ families was too low to evaluate statistically.

**TABLE 2:** Overwintering of *S. bicolor x S. halepense BC_1_F_1_*-derived BC_1_F_2_ families is related to rhizomatousness.

a: 2013-4, Bogart, GA, χ^2^ = 8.84, 1 d.f., *p* = 0.001.		
	**Rhizomes**	**No rhizomes**
Survived	24	1
Died	159	77
**b: 2014-5, Bogart, GA, χ^2^ = 3.10, 1 d.f., *p* = 0.08.**		
Survived	116	28
Died	76	31

More extensive rhizome growth than its rhizomatous diploid progenitor is also related to the ability of *S. halepense* to survive tropical dry seasons. From a total of 96 BC_1_F_2_ families selected for rhizome growth in Bogart GA, single 3 m rows were tested for 15 months (2014-5) at the ICRISAT research station in Samanko, Mali (12.5° N, −7.9° W). A total of 45 (47% of) families contained one or more plants that survived the dry season of 8 month duration with zero rainfall. A logistic regression model (see section “Materials and Methods”) showed that for each 1 cm increase in rhizome spread from the crown based on Bogart GA trials, the probability of surviving the Malian dry season increased ∼3%. Factors other than rhizomes are also important to perenniality – lines surviving the tropical dry season were only randomly associated with those surviving the mild 2014-15 temperate winter in Bogart, GA (24 of 54 lines, 44%), survivors of the harsh 2013-14 winter being more closely associated with dry season survival but too few in overall number to be conclusive (5 of 6, 83%).

### Rhizome Growth Is Correlated With Reproduction

Curiously, rhizome growth is correlated negatively with that of other vegetative organs but positively with reproductive growth. Across four environments (Bogart GA and Salina KS, 2013 and 2014), early flowering is correlated with reduced aboveground vegetative biomass (*r* = −0.26 to −0.62, *p* < 0.001), but increased rhizome growth (*r* = 0.17 to 0.30, *p* < 0.001) in tetraploid *SbxSh* progeny. Because rhizomes are a vegetative organ, our *a priori* expectation was that increased vegetative biomass aboveground would be correlated with increased rhizome growth. However, we measured rhizome growth primarily based on counting above-ground shoots derived from rhizomes. In another rhizomatous grass (*Agropyron repens*), rhizome axillary buds experience apical dominance until anthesis, being suppressed by auxins (Leakey et al., 1975). By excising *S. halepense* rhizomes from the plant, we found that axillary buds consistently develop as vertical shoots and not as rhizomes (Figure 1). So, once flowering of the primary stalk is initiated, a rhizomatous plant permits the development of additional ramets – which in principle should be able to exert apical dominance themselves. Moreover, our observation that these new buds invariably become ramets and not rhizomes raises questions about their additional dependence on a mobile ‘florigen’ such as that translocated to the plant apex (Sachs, 1865). There may be much to be learned about nature of signaling among ramets at different developmental stages that are interconnected by rhizomes.

### Both Polyploidy and Interspecific Hybridity Appear to Contribute to the ‘Mosaic’ Nature of Rhizome Gene Expression

While ∼80% of annotated sorghum genes are expressed in *S. halepense* rhizomes, many alleles with striking enrichment (*p* < 0.001) of expression more closely resemble the sequences of the non-rhizomatous *S. bicolor* progenitor than rhizomatous *Sp*. By laser capture microdissection, we collected meristems and compared transcripts from buds induced to develop as rhizomes or leafy shoots (Figure 1), respectively obtaining 163,264,254 and 152,162,240 Illumina Hiseq reads, of which 67.7% (110,492,577) and 67.2% (102,194,352) could be anchored to 27,566 and 27,183 sorghum gene models. About 1% (262) of genes showed differential expression (*p* < 0.001) between rhizome buds (168 enriched) and shoot buds (94: Supplementary Table 4). Appreciable recruitment of alleles from non-rhizomatous *S. bicolor* to rhizome-enriched expression is indicated by 44 *S. bicolor* versus only 23 *S. propinquum* derived transcripts with at least two SNPs supporting these origins and no contradictory SNPs (other differentially expressed genes are ambiguous based on these criteria).

**FIGURE 2:**
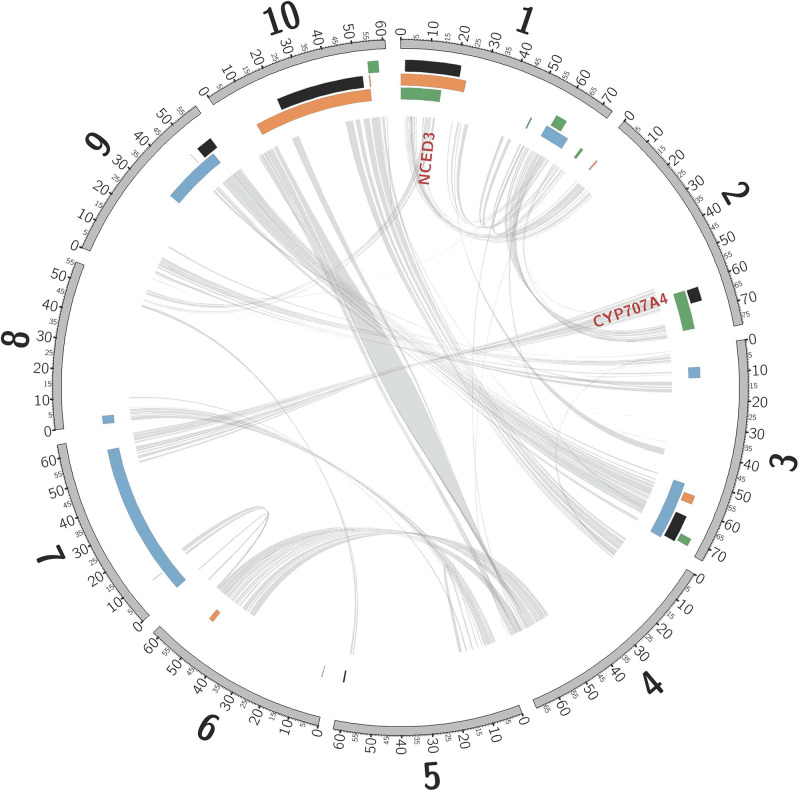
Non-random association between QTLs mapped in sorghum, and introgression from *S. bicolor* into *S. halepense*. Sorghum chromosomes (units are megabases), annotated with physical locations of hotspots of introgression from *S. bicolor* into *S. halepense* [black (Morrell et al., 2005)] and QTLs for seed lutein concentration [green (Fernandez et al., 2008)], seedling vigor [orange (Fernandez et al., 2008)], and rhizomes [blue (Zhang et al., 2013)]. NCED3 and CYP707A4 are gene candidates for cold tolerance (see text). Internal lines indicate syntenic duplicated genes persisting from whole-genome duplication ∼96 million years ago (Paterson et al., 2009; Wang et al., 2015).

Consistent with rhizomes being ∼70% of the mass of a Johnsongrass plant (Oyer et al., 1959), genes highly expressed in rhizome buds were enriched for diverse functions associated with rapid cell division (Kinesins, ATP binding, and microtubule related: Supplementary Table 5) and maturation. Cellulose synthase, Sb06g016760, was the most rhizome enriched gene, also implicated in rapid cell growth. Shoot-bud enriched genes were over-represented in three gene ontology (GO) categories associated with cell recognition (Supplementary Table 5), perhaps in preparation for new biotic interactions after emergence from the soil. The most shoot-enriched genes were (a) glutathione *S*-transferase (Sb09g000860), catalyzing conjugation of the reduced form of glutathione (GSH) to xenobiotic substrates for detoxification; (b) a glycoside hydrolase (Sb08g007610), suggesting cell wall loosening during the rhizome-to-shoot transition; and (c) a member of the major facilitator superfamily (Sb06g033080, MFS: Interpro IPR005828) of transmembrane single-polypeptide secondary carriers implicated in control of sorghum seed size (Zhang et al., 2015), a trait that shows strong negative correlation with both rhizome development and winter survival (TSC, personal communication). Intriguing differentially expressed genes located within likelihood intervals of rhizome related quantitative trait loci (QTLs, Figure 2) include an auxilin/cyclin G-associated kinase (Sb03g028900), tandemly duplicated ethylene responsive transcription factors (Sb07g006195, Sb07g006200), and a Ca2 + /calmodulin-dependent protein kinase, EF-Hand protein superfamily gene (Sb09g022960).

Both polyploidy and interspecific hybridity appear to contribute to the ‘mosaic’ nature of rhizome gene expression, with overexpression of some homoeologs from rhizomatous *S. propinquum* and others from non-rhizomatous *S. bicolor* (Supplementary Table 4). For example, different calmodulin family members have evolved specificity to rhizome buds (e.g., Sb10g027610, the second-most rhizome specific gene) and shoot buds (Sb06g023700). Tandem duplicated ethylene responsive transcription factors within a rhizome-related QTL are both overexpressed in *S. halepense* rhizome buds, although the sequence of Sb07g006195 closely resembles *S. propinquum* (5 of 6 SNPs matching) and adjacent Sb07g006200 is identical to *S. bicolor* (6 of 6 SNPs). The *Teosinte-branched 1* growth repressor gene implicated in apical dominance of maize shoots has two family members with enriched expression in rhizome buds (Sb01g010690, Sb04g026970), ironically both completely matching the non-rhizomatous *S. bicolor* progenitor sequences (4 of 4, and 2 of 2 SNPs).

### Adaptation by *S. halepense* to New Continents and Latitudes May Have Been Facilitated by Introgression From Cultivated Sorghum

Introgression is suggested in a general sense by *S. bicolor* enriched allele composition of the *S. halepense* draft genome (Table 1), and for specific genes by *S. halepense* SNP distribution patterns matching the *S. bicolor* reference genome of an elite breeding line (Paterson et al., 2009), but differing from both several wild *S. bicolors* and each of two outgroups (Table 1, patterns 2–3). Seven ‘hotspots’ for introgression of sorghum alleles in five geographically diverse US *S. halepense* populations (Morrell et al., 2005), show non-random correspondence with published sorghum QTLs (Zhang et al., 2013) conferring variation in rhizome growth, seed size, and lutein content (Figure 2 and Table 3). While sorghum lacks rhizomes and has large seeds, rhizome growth-related alleles masked in domesticated sorghum genotypes by a lack of rhizomes may be unmasked in interspecific crosses with rhizomatous *S. halepense*.

**TABLE 3:** Sorghum QTLs associated with chromosomal ‘hotspots’ for introgression of sorghum alleles into five geographically diverse *S. halepense* populations (Morrell et al., 2005).

Trait	Hotspots	% of genome	# QTLs	Enrichment	*p*-Value (hypergeometric)
Rhizome number	4	13.3	8	4.30	8.82E-05
Seed size	4	8.6	4	6.64	5.14E-04
Lutein	3	5.0	4	8.64	2.58E-03

Particularly intriguing among *S. halepense* introgression hotspots are those that correspond with 3 of 4 QTL likelihood intervals spanning 4.9% of the genome that account for variation in seed content of the carotenoid lutein (Fernandez et al., 2008) (*p* = 0.0026, Table 3). Sorghum leaf photosynthetic capacity is susceptible to damage under low-temperature (<10 C) but high-light conditions when electron transport exceeds the capacity of carbon fixation to utilize available energy (Taylor and Rowley, 1971). Such conditions are infrequent in the tropics where *Sorghum* originated but common in the temperate springtime. Spring regrowth of *S. halepense* starts about 4 weeks before cultivated sorghum is seeded at 38.7° N (Gypsum, KS, where Gypsum 9 was collected). Xanthophyll carotenoids such as lutein are most abundant in plant leaves, modulating light energy and performing non-photochemical quenching of excited ‘triplet’ chlorophyll which is overproduced at very high light levels during photosynthesis (Demmig-Adams and Adams, 2006; Taiz and Zeiger, 2006). Ironically, Sb01g030050 (*Lut1*; KO:K09837) and Sb01g048860 (*crtZ*; KO:K15746) related to lutein biosynthesis, are close to the only lutein QTL not near an introgression hotspot (on chromosome 1).

Within the lutein QTL likelihood intervals, and homozygous in the Gypsum 9E (Supplementary Table 6), are also loss of function mutations in Sb01g013520, 9-*cis* epoxycarotenoid dioxygenase. This enzyme cleaves xanthophylls to xanthoxin, a precursor of the plant hormone abscisic acid (ABA) (Tan et al., 2003) that plays a central role in regulating plant tissue quiescence. Also in the lutein QTL likelihood intervals are non-synonymous SNPs inferred to have striking functional effects (see section “Materials and Methods”) on Sb02g026600, a cytochrome P450 performing a key step of ABA catabolism (Saito et al., 2004). A hypothesis for investigation is whether modified alleles at these loci degrade ABA to release *S. halepense* seeds from dormancy early and/or increase seedling vigor under cold conditions.

## Discussion

Synergy between gene duplication and interspecific hybridity may add an important element to the classical notion that polyploids adapt better than their diploid progenitors to environmental extremes (Muntzing, 1936; Love and Love, 1949; Stebbins, 1950; Grant, 1971). Evidence is growing that polyploidy is an important contributor to biological invasions (te Beest et al., 2012). Genome duplication facilitates the evolution of genes with new or modified functions (Ohno, 1970) such as we report, permitting a nascent polyploid to adapt to environments beyond the reach of its progenitors. Hybridity preserves novel alleles such as many recruited into *S. halepense* rhizome-enriched gene expression from non-rhizomatous *S. bicolor*, putatively contributing to the transgressive rhizome growth and ability of *S. halepense* but not rhizomatous *S. propinquum* derived progeny to overwinter in the temperate United States.

Several lines of evidence point to a richness of DNA-level variation in *S. halepense*, including an abundance of novel coding sequences, much richer diversity of neutral DNA markers than its progenitors, and novel gene expression patterns exemplified by rhizome-enriched expression of some alleles from its non-rhizomatous *S. bicolor* progenitor. The spread of invasive taxa is much more rapid than migration in native taxa, and may require more genetic variation to sustain (Lee, 2002). Although there is somewhat less variation near the invasion front than the center of its US distribution (Sezen et al., 2016), rich *S. halepense* diversity may support its projected 200–600 km northward spread in the coming century (McDonald et al., 2009).

Rich genetic variation in *S. halepense* offers not only challenges but also opportunities. Long under selection for weediness-related attributes that enhance its competitiveness with crops, some US *S. halepense* genotypes have transitioned to non-agricultural niches (Sezen et al., 2016) and may also experience selection favoring alleles that could improve sorghum and other crops, e.g., for cold tolerance, rapid vegetative development and flowering, disease and pest resistance, and ratooning (a new growth cycle from the stubble of the prior one). *Sorghum bicolor* can routinely serve as the pollen parent of triploid and tetraploid (reviewed in Warwick and Black, 1983; Tang and Liang, 1988) and under some circumstances diploid (Dweikat, 2005; Cox et al., 2017), interspecific hybrids with *Sh*, offering the opportunity to test *S. halepense* alleles in sorghum.

As the first surviving polyploid in its lineage in ∼96 million years (Paterson et al., 2009; Wang et al., 2015), *S. halepense* may open new doors to sorghum improvement, with synergy between gene duplication and interspecific hybridity nurturing the evolution of genes with new or modified functions (Ohno, 1970). Already, genetic novelty from *S. halepense* is being used in efforts to breed ratooning/perennial sorghums that better protect ‘ecological capital’ such as topsoil and organic matter (Glover et al., 2010). Attributes of *S. halepense* such as endophytic nitrogen fixation (Rout et al., 2013), if transferred to sorghum, could help to narrow a ‘yield gap’ reflected by 1961–2012 yield gains in the U.S. of only 61% for sorghum versus 323% for maize^2^. Likewise, its perenniality may have resulted in selection for ‘durable’ biotic stress resistance mechanisms that are absent from, but of importance to the improvement of, sorghum and other crops.

## Data Availability Statement

The dataset(s) used in this study can be found as follows: the resequenced genome for tetraploid *S. halepense* accession Gypsum 9 is archived under NCBI ID SRX142088. The resequenced genome for *S. propinquum* is archived under NCBI ID SRX030701-03. The resequenced genome for *Sorghum* [*Sarga* (Hawkins et al., 2015)] *timorense* is archived under NCBI ID SRX124552. LM RNA-seq reads are archived under NCBI BioProject ID PRJNA356741.

## Author Contributions

AP contributed conception and design of the study and wrote the first draft of the manuscript. WK, RJ, PN, VG, KI, US, MK, DB, EW, HR, JB, KB, TC, and MS collected field and/or laboratory data. GW, WP, T-HL, HG, DZ, and FF performed statistical analyses. All authors contributed to manuscript revision, read and approved the submitted version.

## Conflict of Interest

The authors declare that the research was conducted in the absence of any commercial or financial relationships that could be construed as a potential conflict of interest.

## Footnotes

1 http://www.georgiaweather.net/index.php?variable=HI&site=WATUGA

2 http://faostat.fao.org

## References

[B1] Arumuganathan K. Earle E. (1991). Nuclear DNA content of some important plant species. *Plant Mol. Biol. Rep.* 9 208–218. 10.1007/bf02672069

[B2] Binimelis R. Pengue W. Monterroso I. (2009). Transgenic treadmill: responses to the emergence and spread of glyphosate-resistant *Johnsongrass* in argentina. *Geoforum* 40 623–633. 10.1016/j.geoforum.2009.03.009

[B3] Bolger A. M. Lohse M. Usadel B. (2014). Trimmomatic: a flexible trimmer for Illumina sequence data. *Bioinformatics* 30 2114–2120. 10.1093/bioinformatics/btu170 24695404 PMC4103590

[B4] Celarier R. (1958). Cytotaxonomic notes on the subsection *Halepense* of the genus Sorghum. *Bull. Torrey Bot. Club.* 85 49–62.

[B5] Chittenden L. M. Schertz K. F. Lin Y. R. Wing R. A. Paterson A. H. (1994). A detailed RFLP map of sorghum bicolor X S. propinquum, suitable for high-density mapping, suggests ancestral duplication of sorghum chromosomes or chromosomal segments. *Theor. Appl. Genet.* 87 925–933. 10.1007/bf00225786 24190526

[B6] Cox S. Nabukalu P. Paterson A. H. Kong W. Auckland S. Rainville L. (2017). High proportion of diploid hybrids produced by interspecific diploid × tetraploid *sorghum* hybridization. *Genet. Resourc. Crop Evol.* 65 387–390. 10.1007/s10722-017-0580-7

[B7] da Huang W. Sherman B. T. Tan Q. Collins J. R. Alvord W. G. Roayaei J. (2007). The DAVID gene functional classification tool: a novel biological module-centric algorithm to functionally analyze large gene lists. *Genome Biol.* 8:R183. 17784955 10.1186/gb-2007-8-9-r183 PMC2375021

[B8] Demmig-Adams B. Adams W. W. (2006). dams: photoprotection in an ecological context: the remarkable complexity of thermal energy dissipation. *New Phytol.* 172 11–21. 10.1111/j.1469-8137.2006.01835.x 16945085

[B9] Doggett H. (1976). “Sorghum,” in *Evolution of Crop Plants*, ed. Simmonds N. (Essex: Longman).

[B10] Dweikat I. (2005). A diploid, interspecific, fertile hybrid from cultivated sorghum. sorghum bicolor, and the common *Johnsongrass* weed *Sorghum halepense*. *Mol. Breed.* 16 93–101. 10.1007/s11032-005-5021-1

[B11] Feltus F. A. Hart G. E. Schertz K. F. Casa A. M. Brown P. Klein P. E. (2006). Genetic map alignment and QTL correspondence between inter- and intra-specific sorghum populations. *Theor. Appl. Genet.* 112 1295–1305. 10.1007/s00122-006-0232-3 16491426

[B12] Feltus F. A. Wan J. Schulze S. R. Estill J. C. Jiang N. Paterson A. H. (2004). An SNP resource for rice genetics and breeding based on subspecies Indica and Japonica genome alignments. *Genome Res.* 14 1812–1819. 10.1101/gr.2479404 15342564 PMC515328

[B13] Fernandez M. G. S. Hamblin M. T. Li L. Rooney W. L. Tuinstra M. P. Kresovich S. (2008). Quantitative trait loci analysis of endosperm color and carotenoid content in sorghum grain. *Crop Sci.* 48 1732–1743. 10.2135/cropsci2007.12.0684

[B14] Glover J. D. Reganold J. P. Bell L. W. Borevitz J. Brummer E. C. Buckler E. S. (2010). Increased food and ecosystem security via perennial grains. *Science* 328 1638–1639. 10.1126/science.1188761 20576874

[B15] Grant V. (1971). *Plant Speciation*, 1st Edn, New York, NY: Columbia University Press.

[B16] Haas B. J. Papanicolaou A. Yassour M. Grabherr M. Blood P. D. Bowden J. (2013). De novo transcript sequence reconstruction from RNA-seq using the Trinity platform for reference generation and analysis. *Nat. Protoc.* 8 1494–1512. 10.1038/nrot.2013.084 23845962 PMC3875132

[B17] Hawkins J. S. Ramachandran D. Henderson A. Freeman J. Carlise M. Harris A. (2015). Phylogenetic reconstruction using four low-copy nuclear loci strongly supports a polyphyletic origin of the genus Sorghum. *Ann. Bot.* 116 291–299. 10.1093/aob/mcv097 26141132 PMC4512199

[B18] Heap I. (2012). *The International Survey of Herbicide Resistant Weeds.* Ames, IA: Iowa State University Extension and Outreach.

[B19] Kellogg E. (2013). “Phylogenetic relationships of Saccharinae and Sorghinae,” in *Genomics of the Saccharinae.* ed. Paterson A. H. (New York, NY: Springer).

[B20] Kong W. (2017). “Genetic dissection of plant architecture and life history traits salient to climate-resilient sustainable intensification of agriculture,” in *Department of Crop and Soil Science* (Athens, GA: University of Georgia).

[B21] Kong W. Kim C. Goff V. H. Zhang D. Paterson A. H. (2015). Genetic analysis of rhizomatousness and its relationship with vegetative branching of Sorghum bicolor × S. propinquum recombinant inbred lines. *Am. J. Bot.* 102 718–724. 10.3732/ajb.1500035 26022486

[B22] Langmead B. Salzberg S. L. (2012). Fast gapped-read alignment with Bowtie 2. *Nat. Methods* 9 357–359. 10.1038/nmeth.1923 22388286 PMC3322381

[B23] Larkin M. A. Blackshields G. Brown N. P. Chenna R. McGettigan P. A. McWilliam H. (2007). Clustal W and Clustal X version 2.0. *Bioinformatics* 23 2947–2948. 10.1093/bioinformatics/btm404 17846036

[B24] Leakey R. R. B. Chancellor R. J. Vince-Prue D. (1975). Parental factors in dominance of lateral buds on rhizomes of *Agropyron repens* (L.). *Beauv. Plant.* 123 267–274. 10.1007/BF00390705 24435126

[B25] Lee C. E. (2002). Evolutionary genetics of invasive species. *Trends Ecol. Evol.* 17 386–391. 10.1016/s0169-5347(02)02554-5

[B26] Li H. Durbin R. (2009). Fast and accurate short read alignment with burrows-wheeler transform. *Bioinformatics* 25 1754–1760. 10.1093/bioinformatics/btp324 19451168 PMC2705234

[B27] Li H. Handsaker B. Wysoker A. Fennell T. Ruan J. Homer N. (2009). The sequence alignment/map format and SAMtools. *Bioinformatics* 25 2078–2079. 10.1093/bioinformatics/btp352 19505943 PMC2723002

[B28] Love A. Love D. (1949). The geobotanical significance of polyploidy. *Portug. Acta* 5 273–352.

[B29] McDonald A. Riha S. DiTommaso A. DeGaetano A. (2009). Climate change and the geography of weed damage: analysis of US maize systems suggests the potential for significant range transformations. *Agric. Ecosyst. Environ.* 130 131–140. 10.1016/j.agee.2008.12.007

[B30] McWhorter C. G. (1971). Introduction and spread of *Johnsongrass* in the United States. *Weed Sci.* 19:496 10.1017/s0043174500050517

[B31] Morrell P. L. Williams-Coplin D. Bowers J. E. Chandler J. M. Paterson A. H. (2005). Crop-to-weed introgression has impacted allelic composition of *Johnsongrass* populations with and without recent exposure to cultivated sorghum. *Mol. Ecol.* 14 2143–2154. 10.1111/j.1365-294x.2005.02579.x 15910333

[B32] Muntzing A. (1936). The evolutionary significance of autopolyploidy. *Hereditas* 21 363–378. 10.1111/j.1601-5223.1936.tb03204.x

[B33] Ohno S. (1970). *Evolution by Gene Duplication.* Berlin: Springer.

[B34] Oyer E. Gries G. Rogers B. (1959). The seasonal reproduction of johnson grass plants. *Weeds* 7:13 10.2307/4040251

[B35] Paterson A. Schertz K. Lin Y. Liu S. Chang Y. (1995). The weediness of wild plants: molecular analysis of genes influencing dispersal and persistence of *Johnsongrass*. *Sorghum halepense* (L.). *Pers. Proc. Natl. Acad. Sci. U.S.A.* 92 6127–6131. 10.1073/pnas.92.13.6127 11607551 PMC41655

[B36] Paterson A. H. Bowers J. E. Bruggmann R. Dubchak I. Grimwood J. Gundlach H. (2009). The sorghum bicolor genome and the diversification of grasses. *Nature* 457 551–556. 10.1038/nature07723 19189423

[B37] Quinn L. Barney J. N. McCubbins J. Endres A. (2013). Navigating the “Noxious” and “Invasive”. Regulatory landscape: suggestions for improved regulation. *Bioscience* 63 124–131. 10.1525/bio.2013.63.2.8

[B38] Reva B. Antipin Y. Sander C. (2011). Predicting the functional impact of protein mutations: application to cancer genomics. *Nucleic Acids Res.* 39:e118. 10.1093/nar/gkr407 21727090 PMC3177186

[B39] Rout M. E. Chrzanowski T. H. DeLuca T. H. Westlie T. K. Callaway R. M. Holben W. E. (2013). Bacterial endophytes enhance invasive plant competition. *Am. J. Bot.* 100 1726–1737. 10.3732/ajb.1200577 23935109

[B40] Sachs J. (1865). Wirkung des lichtes auf die blütenbildung unter vermittlung der laubblätter. *Bot. Ztg.* 23 117–121.

[B41] Saito S. Hirai N. Matsumoto C. Ohigashi H. Ohta D. Sakata K. (2004). Arabidopsis CYP707As encode (+)-abscisic acid 8′-hydroxylase, a key enzyme in the oxidative catabolism of abscisic acid. *Plany Physiol.* 134 1439–1449. 10.1104/pp.103.037614 15064374 PMC419820

[B42] Sezen U. U. Barney J. N. Atwater D. Z. Pederson G. A. Pedersen J. F. Chandler J. M. (2016). Multi-phase US spread and habitat expansion of a post-columbian invasive. *Sorghum halepense*. *PLoS One* 11:e01644584. 10.1371/journal.pone.0164584 27755565 PMC5068735

[B43] Stebbins G. L. (1950). *Variation and Evolution in Plants.* New York, NY: Columbia University Press.

[B44] Taiz L. Zeiger E. (2006). *Plant Physiology.* Sunderland, MA: Sinauer Associates Inc.

[B45] Takacs E. M. Li J. Du C. Ponnala L. Janick-Buckner D. Yu J. (2012). Ontogeny of the maize shoot apical meristem. *Plant Cell* 24 3219–3234. 22911570 10.1105/tpc.112.099614 PMC3462627

[B46] Tan B. C. Joseph L. M. Deng W. T. Liu L. Li Q. B. Cline K. (2003). Molecular characterization of the *Arabidopsis* 9-cis epoxycarotenoid dioxygenase gene family. *Plant J.* 35 44–56. 12834401 10.1046/j.1365-313x.2003.01786.x

[B47] Tang H. Liang G. H. (1988). The genomic telationship between cultivated sorghum Sorghum bicolor (L) Moench and *johnsongrass* [*Sorghum halepense* (L) Pers] - a reevaluation. *Theor. Appl. Genet.* 76 277–284. 10.1007/bf00257856 24232116

[B48] Taylor A. O. Rowley J. A. (1971). Plants under climatic stress: I. Low temperature, high light effects on photosynthesis. *Plant Physiol.* 47 713–718. 10.1104/pp.47.5.713 16657691 PMC396757

[B49] te Beest M. Roux J. J. Richardson D. M. Brysting A. K. Suda J. Kubešova M. (2012). The more the better? The role of polyploidy in facilitating plant invasions. *Ann. Bot.* 109 19–45. 10.1093/aob/mcr277 22040744 PMC3241594

[B50] Tellman B. (ed.) (1996). “Stowaways and invited guests: how some exotic plants reached the american southwest,” in *California Exotic Pest Council 1996 Symposium*, (San Diego: California Exotic Pest Council).

[B51] Trapnell C. Pachter L. Salzberg S. L. (2009). TopHat: discovering splice junctions with RNA-Seq. *Bioinformatics* 25 1105–1111. 10.1093/bioinformatics/btp120 19289445 PMC2672628

[B52] Trapnell C. Roberts A. Goff L. Pertea G. Kim D. Kelley D. R. (2012). Differential gene and transcript expression analysis of RNA-seq experiments with tophat and cufflinks. *Nat. Protoc.* 7 562–578. 10.1038/nprot.2012.016 22383036 PMC3334321

[B53] Trapnell C. Williams B. A. Pertea G. Mortazavi A. Kwan G. Van Baren M. J. (2010). Transcript assembly and quantification by RNA-Seq reveals unannotated transcripts and isoform switching during cell differentiation. *Nat. Biotechnol.* 28 511–515. 10.1038/nbt.1621 20436464 PMC3146043

[B54] Wang X. Wang J. Guo H. Jin D. Lee T.-H. Liu T. (2015). Genome alignment spanning major Poaceae lineages reveals heterogeneous evolutionary rates and alters inferred dates for key evolutionary events. *Mol. Plant* 8 885–898. 10.1016/j.molp.2015.04.004 25896453

[B55] Warwick S. I. Black L. D. (1983). The biology of canadian weeds. 61. *Sorghum halepense* (L.). *Pers. Can. J. Plant Sci.* 63 997–1014. 10.4141/cjps83-125

[B56] Zhang D. Guo H. Kim C. Lee T. H. Li J. Robertson J. (2013). H.: CSGRqtl, a comparative QTL database for saccharinae grasses. *Plant Physiol.* 161 594–599. 10.1104/pp.112.206870 23370713 PMC3561006

[B57] Zhang D. Li J. Compton R. O. Robertson J. Goff V. H. Epps E. (2015). Comparative genetics of seed size traits in divergent cereal lineages represented by sorghum (Panicoidae) and rice (Oryzoidae). *G3* 3 1117–1128. 10.1534/g3.115.017590 25834216 PMC4478542

