# Supplementary material for: The Evolution of an Invasive Plant, Sorghum halepense L. (‘Johnsongrass’)
Source: Front Genet. 2020 May 14;11:317. doi: 10.3389/fgene.2020.00317 (PMC7240026; doi:10.3389/fgene.2020.00317)
Supplement: Supplementary file 1 [file Presentation_1.pdf]

Supplementary Material

Supplementary Table 1: Coding DNA sequence polymorphism patterns among Sorghum halepense, its progenitors S. propinquum and wild S. bicolor, an elite domesticated S. bicolor, and the outgroup S. timorense. x indicates sequence divergence, o indicates correspondence (data summarized in Table 1).

Table is 196,872 lines long, needs to be uploaded separately.

Supplementary Table 2: Coding DNA sequence polymorphisms with high inferred 'functional impact score' (S_i) suggest important consequences for protein function in 5957 *Sh* genes.

Table comprises 3 sheets, that are up to 5982 lines long, need to be uploaded separately.

Supplementary Table 3:

Function enrichment test of genes containing potential deleterious SNVs that are unique to S. halepense ($P<0.05$).

	Cellular component	Molecular function	Biological process
Premature	Golgi apparatus 163<br>Nucleus 1091<br>Cell wall 112<br>Extracellular 333<br>Plasma membrane 545	Hydrolase activity 448<br>Kinase activity 248<br>Transferase 475<br>Receptor binding 32<br>Nucleotide binding 620	Protein metabolism 679<br>Response to stress 674<br>Response to abiotic or biotic stimulus 633<br>DNA/RNA metabolism 156
Loss of stop codon	Plasma membrane 320	Kinase activity 146<br>Receptor binding 21<br>Nucleotide binding 372	Protein metabolism 387<br>DNA/RNA metabolism 99
Translation initiation	Plasma membrane 33	NA	NA
Total	Extracellular 461<br>Plasma membrane 781	Hydrolase activity 645<br>Kinase activity 347<br>Transferase 669<br>Receptor binding 48<br>Nucleotide binding 873	Protein metabolism 967<br>Developmental process 830<br>DNA/RNA metabolism 231

Supplementary Table 4: Gene expression in *S. halepense* rhizome buds and rhizome-derived shoot buds, and associated statistics, also including *S. bicolor* or *S. propinquum*-associated SNPs.

Table is 99,501 rows, needs to be uploaded separately.

Supplementary Table 5: GO analysis of functions enriched in genes differentially expressed in rhizome buds or shoot buds.

GeneList	Fishers_pVal	bonferroni	benjamini	fdr	efold	Type	Term	Definition
DEG	0.000918	0.186379	0.186379	0.000918	68.10	PFAM	PF00956	Nucleosome assembly protein (NAP)
Rhizome_ON_shoot_OFF	0.000019	0.003992	0.003992	0.000019	1.97	GO	GO:0005524	ATP binding
Rhizome_ON_shoot_OFF	0.000238	0.049485	0.016495	0.000240	7.71	GO	GO:0007018	microtubule-based movement
Rhizome_ON_shoot_OFF	0.000238	0.049485	0.012371	0.000241	7.71	GO	GO:0003777	microtubule motor activity
RhizomeON_shoot_OFF	0.000238	0.049485	0.009897	0.000243	7.71	GO	GO:0005871	kinesin complex
Rhizome_ON_shoot_OFF	0.000065	0.008987	0.004493	0.000066	24.70	KEGG	K10406	KIFC2_3; kinesin family member C2/C3
Rhizome_ON_shoot_OFF	0.000445	0.185842	0.030974	0.000450	6.85	PFAM	PF00225	Kinesin motor domain
Rhizome_ON_shoot_OFF	0.000920	0.384645	0.054949	0.000934	10.96	PFAM	PF07717	Oligonucleotide/oligosaccharide-binding (OB)-fold
Rhizome_ON_shoot_OFF	0.000920	0.384645	0.048081	0.000936	10.96	PFAM	PF04408	Helicase associated domain (HA2)
Rhizome_ON_shoot_OFF	0.000920	0.384645	0.042738	0.000938	10.96	PFAM	PF13646	HEAT repeats
Shoot_ON_Rhizome_OFF	0.000469	0.031869	0.015935	0.000476	11.79	GO	GO:0048544	recognition of pollen
Shoot_ON_Rhizome_OFF	0.000326	0.037187	0.037187	0.000326	13.06	PFAM	PF08276	PAN-like domain
Shoot_ON_Rhizome_OFF	0.000540	0.061514	0.020505	0.000549	11.35	PFAM	PF00954	S-locus glycoprotein domain

Supplementary Table 6. Mutations in carotenoid biosynthesis-related genes within QTL likelihood intervals for variation in seed lutein levels.

Sb01g013520 (chr1: 12577208-12579043)

Function annotation:

NCED3 (NINE-CIS-EPOXYCAROTENOID DIOXYGENASE 3); 9-cis-epoxycarotenoid dioxygenase (*20*)

http://sorghum.riken.jp/morokoshi/Data/Sb01g013520.html

SNP mutations:

chromosome_1 12578413 - C A Sb01g013520 CDS 0 631 631 211 E *

chromosome_1 12578434 - C A Sb01g013520 CDS 0 610 610 204 E *

QTL interval:

chr1: 1-15219573 (QTL effect: color, lutein, β-carotene)

Explanation of gene annotation:

A key regulated step in abscisic acid (ABA) biosynthesis in plants is catalyzed by 9-*cis* epoxycarotenoid dioxygenase (NCED), which cleaves 9-*cis* xanthophylls to xanthoxin, a precursor of ABA.

Sb02g026600 (chr2: 61762825-61765740)

Function annotation:

(+)-abscisic acid 8\'-hydroxylase/ oxygen binding

http://sorghum.riken.jp/morokoshi/Data/Sb02g026600.html

SNP mutations:

Sb02g026600 contains several large effect nsSNP and also contains one SNP in intron that is differed between wild SB and domesticated SB.

chromosome_2 61764702 - T G Sb02g026600 Intron 246 1039 1861 . . .

QTL interval:

chr2: 61367786-76144244 (QTL effect: lutein, β-carotene, total carotenoids)

Explanation of gene function (cold tolerance?):

The plant hormone abscisic acid (ABA) regulates many important physiological and developmental processes in plants as well as adaptive responses to environmental stresses. During seed development and dormancy, ABA content increases as a potential signal to organize expression of many embryo-specific genes. The ABA level is also elevated in response to various environmental stresses such as drought, high salinity, and low temperature conditions. The hydroxylation at the 8′-position of ABA is known as the key step of ABA catabolism, and this reaction is catalyzed by ABA 8′-hydroxylase, a cytochrome P450.

7. Mutations in carotenoid biosynthesis-related genes within QTL likelihood intervals for variation in seed lutein levels.

Sb01g013520 (chr1: 12577208-12579043)

Function annotation:

NCED3 (NINE-CIS-EPOXYCAROTENOID DIOXYGENASE 3); 9-cis-epoxycarotenoid dioxygenase (*20*)

http://sorghum.riken.jp/morokoshi/Data/Sb01g013520.html

SNP mutations:

chromosome_1 12578413 - C A Sb01g013520 CDS 0 631 631 211 E *

chromosome_1 12578434 - C A Sb01g013520 CDS 0 610 610 204 E *

QTL interval:

chr1: 1-15219573 (QTL effect: color, lutein, β-carotene)

Explanation of gene annotation:

A key regulated step in abscisic acid (ABA) biosynthesis in plants is catalyzed by 9-*cis* epoxycarotenoid dioxygenase (NCED), which cleaves 9-*cis* xanthophylls to xanthoxin, a precursor of ABA.

Sb02g026600 (chr2: 61762825-61765740)

Function annotation:

(+)-abscisic acid 8\'-hydroxylase/ oxygen binding

http://sorghum.riken.jp/morokoshi/Data/Sb02g026600.html

SNP mutations:

Sb02g026600 contains several large effect nsSNP and also contains one SNP in intron that is differed between wild SB and domesticated SB.

chromosome_2 61764702 - T G Sb02g026600 Intron 246 1039 1861 . . .

QTL interval:

chr2: 61367786-76144244 (QTL effect: lutein, β-carotene, total carotenoids)

Explanation of gene function (cold tolerance?):

The plant hormone abscisic acid (ABA) regulates many important physiological and developmental processes in plants as well as adaptive responses to environmental stresses. During seed development and dormancy, ABA content increases as a potential signal to organize expression of many embryo-specific genes. The ABA level is also elevated in response to various environmental stresses such as drought, high salinity, and low temperature conditions. The hydroxylation at the 8′-position of ABA is known as the key step of ABA catabolism, and this reaction is catalyzed by ABA 8′-hydroxylase, a cytochrome P450.
